# Retrospective analysis of the possibility of predicting the COVID-19 outbreak from Internet searches and social media data, China, 2020

**DOI:** 10.2807/1560-7917.ES.2020.25.10.2000199

**Published:** 2020-03-12

**Authors:** Cuilian Li, Li Jia Chen, Xueyu Chen, Mingzhi Zhang, Chi Pui Pang, Haoyu Chen

**Affiliations:** 1 Joint Shantou International Eye Center, Shantou University and the Chinese University of Hong Kong, Shantou, China; 2 Department of Ophthalmology and Visual Sciences, the Chinese University of Hong Kong, Hong Kong, China

**Keywords:** COVID-19, coronavirus, Internet surveillance, Google Trends, Baidu Index, Weibo Index

## Abstract

The peak of Internet searches and social media data about the coronavirus disease 2019 (COVID-19) outbreak occurred 10–14 days earlier than the peak of daily incidences in China. Internet searches and social media data had high correlation with daily incidences, with the maximum r > 0.89 in all correlations. The lag correlations also showed a maximum correlation at 8–12 days for laboratory-confirmed cases and 6–8 days for suspected cases.

The coronavirus disease 2019 (COVID-19) outbreak began in Wuhan, China, in late December 2019 and quickly spread to other cities in China in a matter of days [1,2]. It was announced as a public health emergency of international concern by the World Health Organization (WHO) on 30 January 2020 [3]. Predicting the development of the outbreak as early and as reliably as possible is critical for action to prevent its spread. Internet searches and social media data have been reported to correlate with traditional surveillance data and can even predict the outbreak of disease epidemics several days or weeks earlier [4-9]. 

In this study, we aimed to evaluate the prediction value of the Internet search data from web-based search engines and social media for the COVID-19 outbreak in China.

## Trends in daily laboratory-confirmed and suspected COVID-19 cases and Internet data

The daily numbers of new laboratory-confirmed cases and suspected cases of COVID-19 were collected from the data published by the National Health Commission of China (NHC, http://www.nhc.gov.cn/). A laboratory-confirmed case of COVID-19 was defined a patient with positive real-time RT-PCR to SARS-CoV-2, while a suspected case was defined as a patient with history of travelling to Wuhan City or in contact with COVID-19 cases in the 14 days before onset of symptoms and with clinical manifestation of fever, respiratory illness, pneumonia on computed tomography (CT) scan, and/or reduced white blood cells count, but no RT-PCR results. The study period was set between 16 January and 11 February 2020, because the diagnosis criteria were set on 16 January 2020. The results showed that the peak of daily new laboratory-confirmed cases was 3,887 on 4 February and the peak of daily new suspected cases was 5,328 on 5 February 2020.

Daily trend data related to specific search terms were acquired from Google Trends, Baidu Index, and Sina Weibo Index by setting the time parameter to ‘2 January to 12 February 2020’ and the location parameter to ‘China’. We chose a period 2 weeks earlier than for the molecular diagnosis data for COVID-2019. Two keywords, ‘coronavirus’ and ‘pneumonia’, were used in Google Trends. The respective Chinese terms, ‘冠状病毒‘ and ‘肺炎’ were used in Baidu Index, the most popular web search engine in China, and Sina Weibo Index, a social media platform widely used in China. The peak number of search queries in Baidu was 682,888 for ‘coronavirus’ and 760,460 for ‘pneumonia’, both on 25 January 2020. The peak number of posts on Sina Weibo was 26,297,746 for ‘coronavirus’ and 30,704,753 for ‘pneumonia’, both on 21 January 2020. Google Trends does not provide the raw number of search queries but the number normalised to the peak number. The peaks for both keywords on Google Trends were reached on 25 January 2020.

**Figure 1:**
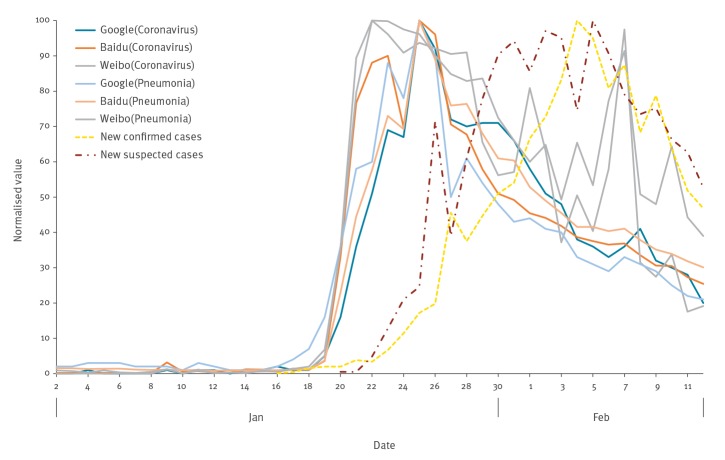
Searches for keywords ‘coronavirus’ and ‘pneumonia’, obtained via different indices, and number of daily new COVID-19 cases, China, January–February 2020

Figure 1 shows the overall trends of data from the keyword search for ‘coronavirus’ (or ‘冠状病毒’) and ‘pneumonia’ (or ‘肺炎’) via Google Trends, Baidu Index and Sina Weibo Index, and the number of daily new laboratory-confirmed and suspected COVID-19 cases. The data from Baidu Index, Sina Weibo Index and national COVID-19 daily incidence data were also normalised to the peak number, so that the values fall into the same range (0–100) during that period.

## Lag correlation between daily laboratory-confirmed/suspected cases and Internet searches

**Figure 2:**
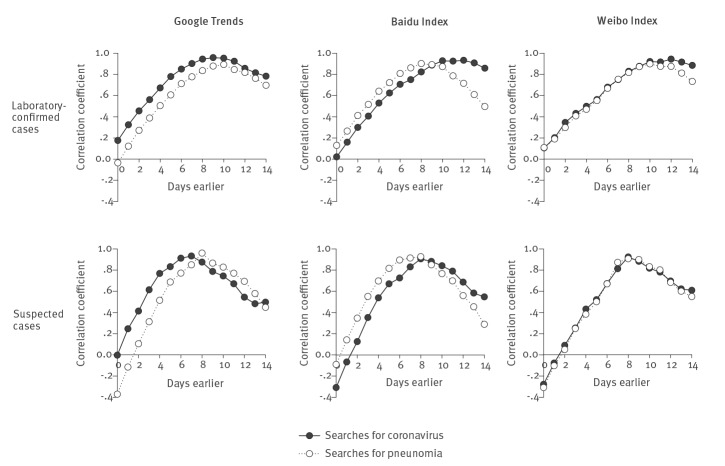
Lag correlations between new laboratory-confirmed cases and suspected cases of COVID-19 and data from Google Trends, Baidu Index and Weibo Index for the keywords ‘coronavirus’ and ‘pneumonia’, China, January–February 2020

Figure 2 and the Table showed the lag Spearman correlations between the daily new laboratory-confirmed cases (upper panel) and suspected cases (lower panel) of COVID-19 and the Internet search data from Google Trends, Baidu Index and Sina Weibo Index. We found a high correlation with the Internet search data (r > 0.7) 8–10 days earlier for new laboratory-confirmed cases, and 5-7 days earlier for new suspected cases.

**Table:** Lag correlation coefficients and p values between Internet search data and daily new laboratory-confirmed/suspected COVID-19 cases, China, January–February 2020

	Days earlier	Google Trends				Baidu Index				Sina Weibo Index			
		Coronavirus	p	Pneumonia	p	Coronavirus	p	Pneumonia	p	Coronavirus	p	Pneumonia	p
New laboratory-confirmed cases	0	0.176	0.370	−0.035	0.861	0.021	0.917	0.129	0.513	0.106	0.593	0.109	0.582
	1	0.324	0.093	0.122	0.537	0.160	0.416	0.265	0.172	0.202	0.303	0.190	0.332
	2	0.455	0.015	0.271	0.164	0.299	0.122	0.411	0.030	0.346	0.072	0.298	0.123
	3	0.561	0.002	0.388	0.041	0.406	0.032	0.516	0.005	0.431	0.022	0.408	0.031
	4	0.672	< 0.001	0.505	0.006	0.529	0.004	0.641	< 0.001	0.498	0.007	0.470	0.012
	5	0.779	< 0.001	0.606	0.001	0.624	< 0.001	0.722	< 0.001	0.562	0.002	0.553	0.002
	6	0.850	< 0.001	0.712	< 0.001	0.706	< 0.001	0.808	< 0.001	0.679	< 0.001	0.668	< 0.001
	7	0.902	< 0.001	0.777	< 0.001	0.750	< 0.001	0.861	< 0.001	0.751	< 0.001	0.754	< 0.001
	8	0.944	< 0.001	0.835	< 0.001	0.823	< 0.001	***0.902***	* * ***< 0.001***	0.829	< 0.001	0.817	< 0.001
	9	***0.958***	* * ***< 0.001***	0.878	< 0.001	0.887	< 0.001	0.892	< 0.001	0.876	< 0.001	0.872	< 0.001
	10	0.953	< 0.001	***0.893***	* * ***< 0.001***	0.928	< 0.001	0.873	< 0.001	0.921	< 0.001	***0.899***	* * ***< 0.001***
	11	0.924	< 0.001	0.845	< 0.001	0.925	< 0.001	0.786	< 0.001	0.917	< 0.001	0.875	< 0.001
	12	0.857	< 0.001	0.818	< 0.001	***0.933***	* * ***< 0.001***	0.715	< 0.001	***0.944***	* * ***< 0.001***	0.875	< 0.001
	13	0.815	< 0.001	0.762	< 0.001	0.908	< 0.001	0.609	0.001	0.916	< 0.001	0.812	< 0.001
	14	0.783	< 0.001	0.697	< 0.001	0.858	< 0.001	0.496	0.007	0.885	< 0.001	0.733	< 0.001
New suspected cases	0	−0.003	0.989	−0.372	0.073	−0.309	0.142	−0.091	0.671	−0.279	0.187	−0.309	0.142
	1	0.246	0.246	−0.116	0.590	−0.068	0.753	0.141	0.511	−0.078	0.716	−0.103	0.630
	2	0.413	0.045	0.104	0.630	0.125	0.560	0.346	0.098	0.089	0.680	0.050	0.818
	3	0.614	0.001	0.312	0.138	0.352	0.091	0.551	0.005	0.253	0.233	0.248	0.243
	4	0.768	< 0.001	0.514	0.010	0.538	0.007	0.697	< 0.001	0.431	0.035	0.383	0.065
	5	0.832	< 0.001	0.687	< 0.001	0.670	< 0.001	0.816	< 0.001	0.520	0.009	0.501	0.013
	6	***0.912***	* * ***< 0.001***	0.771	< 0.001	0.725	< 0.001	0.895	< 0.001	0.672	< 0.001	0.670	< 0.001
	7	0.933	< 0.001	0.850	< 0.001	0.830	< 0.001	0.914	< 0.001	0.813	< 0.001	0.872	< 0.001
	8	0.875	< 0.001	***0.960***	* * ***< 0.001***	***0.906***	* * ***< 0.001***	***0.926***	* * ***< 0.001***	***0.924***	* * ***< 0.001***	***0.907***	* * ***< 0.001***
	9	0.787	< 0.001	0.865	< 0.001	0.882	< 0.001	0.850	< 0.001	0.883	< 0.001	0.899	< 0.001
	10	0.744	< 0.001	0.827	< 0.001	0.841	< 0.001	0.766	< 0.001	0.818	< 0.001	0.832	< 0.001
	11	0.671	< 0.001	0.770	< 0.001	0.790	< 0.001	0.698	< 0.001	0.781	< 0.001	0.802	< 0.001
	12	0.544	0.006	0.693	< 0.001	0.686	< 0.001	0.559	0.005	0.697	< 0.001	0.683	< 0.001
	13	0.482	0.017	0.578	0.003	0.583	0.003	0.454	0.026	0.622	0.001	0.600	0.002
	14	0.497	0.013	0.448	0.028	0.547	0.006	0.288	0.173	0.609	0.002	0.550	0.005

Shaded text: high correlation with r > 0.7. Text in italics: highest correlation.

For new laboratory-confirmed cases, the highest correlation was found 9, 12 and 12 days earlier for searches for the keyword ‘coronavirus’ in Google Trends, Baidu Index and Sina Weibo Index with, respectively, r = 0.958, 0.933 and 0.944. For the keyword ‘pneumonia’, the highest correlation was found 10, 8 and 10 days earlier in Google Trends, Baidu Index and Sina Weibo Index, with r = 0.893, 0.944 and 0.899, respectively.

The lag correlation of new suspected cases was similar to the laboratory-confirmed cases, with a shorter lag time. The highest correlation was found 6, 8 and 8 days earlier for searches for the keyword ‘coronavirus’ in Google Trends, Baidu Index and Sina Weibo Index, with r = 0.912, 0.906 and 0.924, respectively. For the keyword ‘pneumonia’, the highest correlation was found all 8 days earlier in Google Trends, Baidu Index and Sina Weibo Index, with r = 0.960, 0.926 and 0.907, respectively.

## Discussion

Our study demonstrated that the data obtained from Google Trends, Baidu Index and Sina Weibo Index on searches for the keywords ‘coronavirus’ and ‘pneumonia’ correlated with the published NHC data on daily incidence of laboratory-confirmed and suspected cases of COVID-19, with the maximum r > 0.89. We also found that the peak interest for these keywords in Internet search engines and social media data was 10–14 days earlier than the incidence peak of COVID-19 published by the NHC. The lag correlation showed a maximum correlation at 8–12 days for laboratory-confirmed cases and 6–8 days for suspected cases.

COVID-19 is a rapidly spreading infectious disease with, at the time of submission, more than 80,000 cases and a mortality so far known to be 3.4% [10]. It is important to predict the development of this outbreak as early and as reliably as possible, in order to take action to prevent its spread. Our data showed that the two popularly used Internet search engines, Google and Baidu, and the social media platform, Sina Weibo, were able to predict the disease outbreak 1–2 weeks earlier than the traditional surveillance systems. The role of Internet surveillance tools in early prediction of other epidemics has been reported previously, including for influenza [4], dengue fever [5], H1N1 [6], Zika [7], measles [8] and Middle East respiratory syndrome [9]. The availability of early information about infectious diseases through Internet search engines and social media will be helpful for making decisions related to disease control and prevention.

Internet search data have been shown to enable the monitoring of Middle East respiratory syndrome 3 days before laboratory confirmations [9]. However, our results showed a much longer lag time for reported new laboratory-confirmed and suspected COVID-19 cases compared with digital surveillance data. There are several explanations. Firstly, COVID-19 is a novel disease just recently recognised. The first version of a guideline for diagnosis and management of COVID-19 was announced on 16 January 2020. It took time for the medical professionals to learn about the virus and the disease in order to make correct diagnosis. Secondly, the diagnosis of COVID-19 requires two independent confirmatory laboratory tests, which should be taken at least 1 day apart. Our results showed that the lag correlation is shorter for the suspected than for laboratory-confirmed cases. Thirdly, the supply of laboratory testing kits may have been insufficient in the early stages of the coronavirus outbreak, which would have limited the number of patients that can be confirmed. Finally, the Internet searches and social media mentions are not only initiated by the patients and their family members, but also globally by the general public who are concerned about this rapidly spreading disease. 

In addition, we found that the data from the Baidu Index and Sina Weibo Index could monitor the number of daily new confirmed and suspected cases from the NHC earlier than the data from Google Trends. A possible explanation is that the Google is not a major search engine used in China, where Baidu and Sina Weibo are widely used. The peak in the Sina Weibo Index was reached earlier than in Google Trends and Baidu Index. This suggests that Sina Weibo, which also serves as a social medium, disseminated the information faster than traditional websites.

COVID-19 was firstly reported as ‘pneumonia of unknown aetiology’ or ‘pneumonia of unknown cause’ in late December 2019. On 8 January 2020, a novel coronavirus was identified as the cause of this disease. The disease was first named Novel coronavirus pneumonia by the NHC of China on 8 February and later ‘coronavirus disease 2019’ (abbreviated ‘COVID-19’) on 11 February by the WHO. Our search period was defined from January 16 to February 11. Therefore, we think that the two keywords ‘pneumonia’ and ‘coronavirus’ were sufficient to include most Internet content related to COVID-19 in this period. We also used other terms such as ‘新冠‘ (novel coronavirus), ‘新型冠状病毒肺炎’ (novel coronavirus pneumonia) as keywords but they returned much smaller numbers of queries and posts and we did therefore not include them in the analysis.

It is also notable that the strength of correlation was different for different keywords. On Google, the keyword ‘coronavirus’ had the highest correlation coefficient (r = 0.958) with daily new laboratory-confirmed cases, and ‘pneumonia’ had the highest correlation coefficient with daily new suspected cases (r = 0.960). We found the same pattern in the Baidu Index and Sina Weibo Index. An explanation could be that ‘coronavirus’ is linked to the viral pathogen which should be investigated by a laboratory test, while ‘pneumonia’ is a clinical term and should link stronger to the suspected cases that are based on clinical and imaging evidence.

A limitation of our study is its retrospective nature. If the Internet search engines and social media data were used in a real-time surveillance system, finding the best lag time would be a challenge because we would not have any training data to calibrate the analysis for a new disease.

## Conclusion

This study reveals the advantages of Internet surveillance using Sina Weibo Index, Google Trends and Baidu Index to monitor a new infectious disease. Reliable data can be obtained early at low cost. The Internet surveillance data provided an accurate and timely prediction about the outbreak and progression of COVID-19.
